# Radiation-Induced Grafting with One-Step Process of Waste Polyurethane onto High-Density Polyethylene

**DOI:** 10.3390/ma9010013

**Published:** 2015-12-29

**Authors:** Jong-Seok Park, Youn-Mook Lim, Young-Chang Nho

**Affiliations:** Radiation Research Division for Industry and Environment, Korea Atomic Energy Research Institute, 1266 Sinjeong-dong, Jeongeup-si, Jeollabuk-do 580-185, Korea; ymlim71@kaeri.re.kr (Y.-M.L.); jaspa@naver.com (Y.-C.N.)

**Keywords:** waste, polyurethane, radiation grafting, recycling, polyethylene

## Abstract

The recycling of waste polyurethane (PU) using radiation-induced grafting was investigated. The grafting of waste PU onto a high-density polyethylene (HDPE) matrix was carried out using a radiation technique with maleic anhydride (MAH). HDPE pellets and PU powders were immersed in a MAH-acetone solution. Finally, the prepared mixtures were irradiated with an electron beam accelerator. The grafted composites were characterized by Fourier transformed infrared spectroscopy (FT-IR), surface morphology, and mechanical properties. To make a good composite, the improvement in compatibility between HDPE and PU is an important factor. Radiation-induced grafting increased interfacial adhesion between the PU domain and the HDPE matrix. When the absorbed dose was 75 kGy, the surface morphology of the irradiated PU/HDPE composite was nearly a smooth and single phase, and the elongation at break increased by approximately three times compared with that of non-irradiated PU/HDPE composite.

## 1. Introduction

Polyurethane (PU) has been used in a variety of applications due to its good mechanical, thermal, and chemical properties. However, the use of PU has led to the environmental issues because the greater part of the industrial waste PU are being disposed in landfill sites. Thus, research has focused on alternative options for the disposal of PU [1,2], e.g., the recycling or modification of PU waste into useful materials [3].

Polymer blending technology is a promising approach for the ecological and economical remediation of waste plastics [4]. Waste PU can be recycled and used as filler for commodity polymers to improve thermal and mechanical properties [5]. A high compatibility between the commodity polymers and waste PU is an important factor to consider in the formation of a good composite. However, PU is hydrophilic, whereas most commodity polymers are hydrophobic [4,6].

**Scheme 1:**
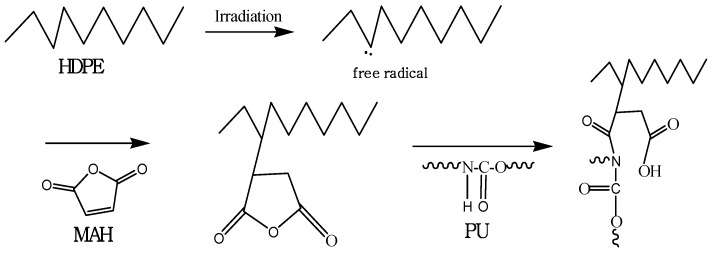
Radiation-induced grafting polymerization.

Graft polymerization is an excellent way to improve the miscibility between PU and commodity polymers [7]. Furthermore, radiation-induced grafting techniques provide several advantages over classical chemical grafting, including uniform grafting compositions, room temperature processing, and initiator and catalyst-free processing [3,8,9]. Radiation-induced grafting can be proceeded between monomer and polymer during the irradiation. The first step is the irradiation to generate polymer radicals and second step is the graft polymerization of monomer on to the polymer through radical polymerization (Scheme 1) [10].

Recently, maleic anhydride (MAH) has become an attractive monomer to improve the hydrophilicity of polyolefin [11]. Polymers grafted with maleic anhydride (MAH) have been used as compatibilizers to improve the interfacial interaction between immiscible polymers [6,12].

Our previous work [13] presented on the polyethylene-graft-maleic anhydride (PE-g-MA) grafted PU/HDPE composite via melt processing in a blender after radiation-induced grafting of PU with PE-g-MA. This process was performed with two-step process. The PE-g-MA easily reacted with the PU according to increasing radiation doses and was located at the interface between the PU and the HDPE during the melt processing in the blender, which improved the interfacial interactions and the mechanical properties of the resultant composites. However, the elongation at break for PU contents >2 phr drastically decreased [13].

The main objective of this study is to develop an effective recycling method of industrial waste PU by radiation-induced grafting. In this study, the radiation grafting of waste PU onto an HDPE matrix was carried out with MAH. HDPE pellets were immersed in MAH-acetone solution, and waste PU powders were added. The prepared mixtures were graft-irradiated with an electron beam accelerator. The grafted composites were characterized by Fourier transformed infrared spectroscopy (FT-IR), surface morphology, and mechanical properties.

## 2. Experimental

### 2.1. Materials

Methylene diphenyl diisocyanate (MDI)-based polyurethane (PU) foam, yellowish in color, was collected from the waste disposals of a railway system from Envista, Inc. (Chungju, Korea). The rigid PU foams were reduced to powder with a knife cutter. Commercial-grade high-density polyethylene (HDPE; 5305E) was used throughout this study and was supplied by Lotte Chemical Corporation (Yeosu, Korea). Maleic anhydride (MAH) was purchased from Sigma Aldrich (St. Louis, MO, USA). All other chemicals used were of reagent grade and were applied as purchased without further purification.

### 2.2. Radiation-Induced Grafting

HDPE pellets were exposed to a gamma ^60^Co source at a total dose of 50 kGy at 10 kGy/h for oxidization. The surface modification of HDPE was carried out by grafting MAH using a radiation technique. MAH powder (10 wt %) was dissolved completely in an acetone solution at room temperature. Then, irradiated HDPE pellets and waste PU powder (0–10 phr) were immersed in the MAH-acetone solution at an HDPE:MAH-acetone solution ratio of 5:2. The unit “phr” is the abbreviation of parts per hundred resin, and the base resin used here is HDPE. They were stirred using a mechanical stirrer for 1 h. The prepared mixtures were irradiated with an electron beam accelerator (10 MeV/1 mA, Jeongup site of KAERI, Jeonbuk, Korea). The total absorbed dose ranged from 25 to 75 kGy. Thereafter, to remove the remaining solvent and moisture, the irradiated mixtures were washed three times with deionized water (DIW) and dried in a vacuum oven at 50 °C for 24 h.

### 2.3. Preparation HDPE/PU Composite

The irradiated HDPE/PU mixtures were melt-blended in a lab-scale blender (Brabender D-47055, Brabender, Duisburg, Germany) with a screw speed of 50 rpm, at 160 °C for 15 min. After blending, the mixtures were prepared using square shaped sheets by hot-press molding at 180 °C for 10 min. The size of the specimens was 100 mm × 100 mm, the thickness was 2 mm.

### 2.4. Characterization of PU/HDPE Composite

The chemical structure of HDPE/PU composites with radiation-induced grafting was confirmed by Fourier transformed infrared spectroscopy (FT-IR; Bruker Tensor 37, Bruker, MA, USA). FT-IR spectra were recorded in the range 2000 to 600 cm^−1^ at a resolution of 4 cm^−1^ in attenuated total reflectance (ATR) mode.

The tensile strength and elongation at break was measured with universal testing machine (Model 4210, Instron Engineering Co., Canton, MA, USA) according to the ASTM Standard D 638 [14]. The size of the specimens was 5 mm *×* 20 mm, the thickness was 2 mm, and the head speed was 50 mm/min.

The dynamic mechanical properties were investigated with a dynamic mechanical analyzer (DMA; DMA Q800, TA Instruments, New Castle, DE, USA), which was varied from 25 to 130 °C at 1 Hz at a heating rate of 5 °C/min.

To observe the high-resolution images, the samples were covered with a layer of osmium (Os) for 60 s by sputter coating. The surface morphology of the HDPE/PU were investigated with a field emission scanning electron microscope (FE-SEM S-4700, Hitachi, Tokyo, Japan) at a resolution of 60 Å at 5 kV, with a magnification of 5.0 K and a working distance of 10–12 mm.

The thermal properties of the HDPE/PU composites were analyzed using a differential scanning calorimetry (DSC) (TA Q100, TA Instrument). The thermograms were recorded from room temperature to 180 °C at a rate of 10 °C/min under N_2_ gas. The degree of crystallinity (*X*c) of HDPE composite can be calculated by the following equation:

(1) $$X\text{c}(\%)=\frac{\Delta H_{\text{m}}}{\Delta H_{\text{m}}^{\text{o}}}\times 100$$

where $\Delta H_{\text{m}}^{\text{o}}$ = 290 J/g is the fusion enthalpy for a totally crystalline HDPE and $\Delta {Η}_{\text{m}}$ is the fusion enthalpy calculated from the area of the endothermic melting peak.

## 3. Results and Discussion

The immiscible properties of PU and HDPE presents a significant challenge in incorporating PU onto an HDPE matrix. PU is hydrophilic, whereas HDPE is hydrophobic. Any blends of these two polymers are generally immiscible. Thus, the interfacial compatibility between PU and an HDPE matrix must be resolved to obtain good mechanical properties [6].

In our previous work, the radiation-induced grafting was carried out with two-step process. The waste PU and PE-g-MA were dissolved in a DMF solution and irradiated to graft using an electron beam accelerator. The PE-g-MA-grafted PU and HDPE were blended using a lab-scale blender. The PU could be easily introduced to the HDPE during the melt processing in the blender after the radiation-induced grafting of PU with PE-g-MA. PE-g-MA was easily reacted with PU according to the increasing radiation dose. However, the elongation at break for a PU content >2 phr was drastically decreased [13].

In this study, the surface modification of HDPE and PU was simultaneously progressed with one-step process by grafting MAH using a radiation technique. The HDPE pellets and waste PU powders were immersed in a MAH-acetone solution. The prepared mixtures were irradiated to graft using an electron beam accelerator. Finally, the irradiated HDPE/PU mixtures were melt-blended in a lab-scale blender.

**Figure 1:**
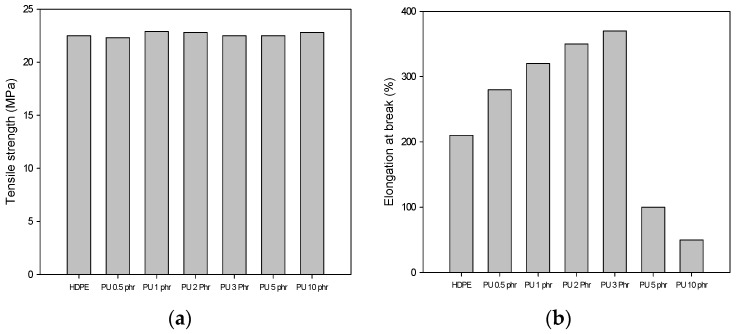
(**a**) Tensile strength; and (**b**) elongation at break of polyurethane/high-density polyethylene (PU/HDPE) composite at various content of PU; radiation dose is 25 kGy.

Figure 1 shows the mechanical properties, including tensile strength and elongation at break of the PU/HDPE composites at various content of PU. In this experiment, the radiation dose was 25 kGy. No changes in the tensile strength were observed in the PU/HDPE composites with increasing concentrations of PU. However, the elongation at break of the PU/HDPE composites increased with increasing contents up to 3 phr. When the content of PU was 3 phr, the elongation at break reached a maximum average value of 380%. The elongation at break increased by approximately four times compared with that of our previous work. The maximum elongation at break of the PU/HDPE composites in our previous work was 280%, when the content of PU was 1 phr. This results indicates a significant improvement in the compatibility between PU and HDPE compared to our previous work [13]. This result is probably that one-step process promoted grafting through the reactions of the peroxy groups of the oxidized HDPE with the carbon double bonds of MAH, and through the carbonyl groups of MAH with the amino groups of PU during the radiation processing [4,11].

**Figure 2:**
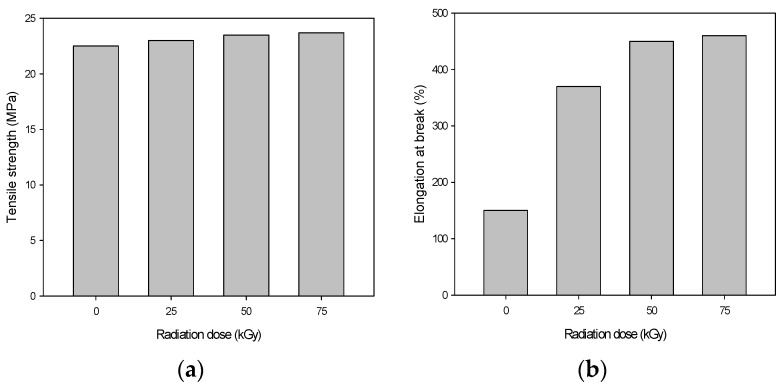
(**a**) Tensile strength; and (**b**) elongation at break of PU (3 phr)/HDPE composite at various absorption doses.

Figure 2 shows the mechanical properties of the PU/HDPE composites at various absorption doses. During this experiment, the content of the PU dose was 3 phr. The tensile strength was tested for each sample at least five times, and the average value at the maximum point was recorded. The tensile strength slightly increased as the radiation dose increased. As shown in Figure 2, the tensile strength at a 75 kGy irradiation dose was 24 MPa. However, the elongation at break drastically increased when the absorbed dose increased. When the absorbed dose was 75 kGy, the elongation at break of the PU/HDPE composite was 460% and increased by approximately three-fold over that of the non-irradiated PU/HDPE composite. This result indicates a significant improvement in the interfacial state between the HDPE and the PU after the radiation-induced grafting of PU.

**Figure 3:**
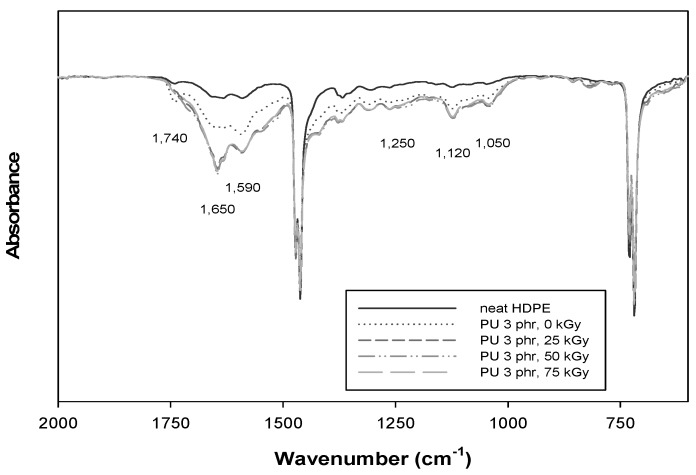
Fourier transformed infrared spectroscopy (FT-IR) spectra of neat HDPE and irradiated PU (3 phr)/HDPE composite at various absorption doses.

The chemical changes of PU/HDPE composites after radiation-induced grafting at various absorbed doses were investigated by FT-IR spectroscopy. Figure 3 shows the FT-IR spectra of the neat HDPE and irradiated PU/HDPE composite at various absorption doses from 2000 to 600 cm^−1^. As shown in the non-irradiated PU/HDPE composite spectra, the typical carbonyl group of maleic anhydride was observed at 1740 cm^−1^. However, the intensity of this peak was decreased significantly as the absorption dose increased. The IR absorption peaks of PU correspond to carbonyl vibrations at 1650 cm^−1^ (C=O stretching). Bending vibrations at 1590 cm^−1^ (N–H bending) were clearly observed [15]. In addition, the PU signal in the irradiated PU/HDPE composites revealed characteristic bands at 1250 cm^−1^ (C–N stretching), 1120 cm^−1^ (C=O stretching and O–CH_2_ stretching), and 1050 cm^−1^ (C–O stretching) [16]. These results demonstrated the successful radiation induced grafting of PU onto an HDPE matrix.

Further evidence of the successful radiation-induced grafting were confirmed by SEM.

**Figure 4:**
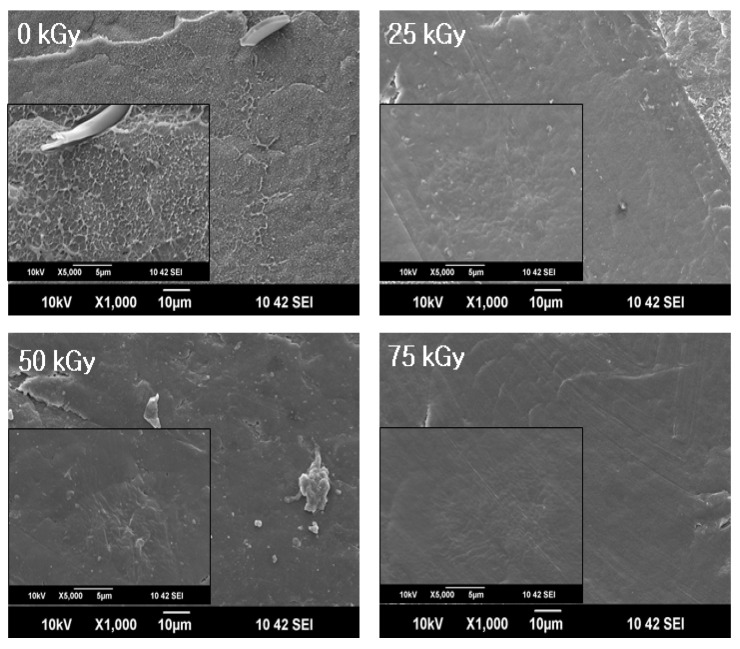
Scanning electron microscope (SEM) micrographs of PU (3 phr)/HDPE composites at various absorption doses.

Figure 4 shows the surface morphologies of the PU/HDPE composites at various absorption doses. The large domains of PU at 0 and 25 kGy were irregularly distributed in the HDPE matrix due to weak interfacial bonding. However, higher irradiation doses (*i.e.*, 50 and 75 kGy) promoted interfacial adhesion between the PU domain and the HDPE matrix. When the absorbed dose was 75 kGy, the surface morphologies were nearly smooth and consisted of a single phase.

**Figure 5:**
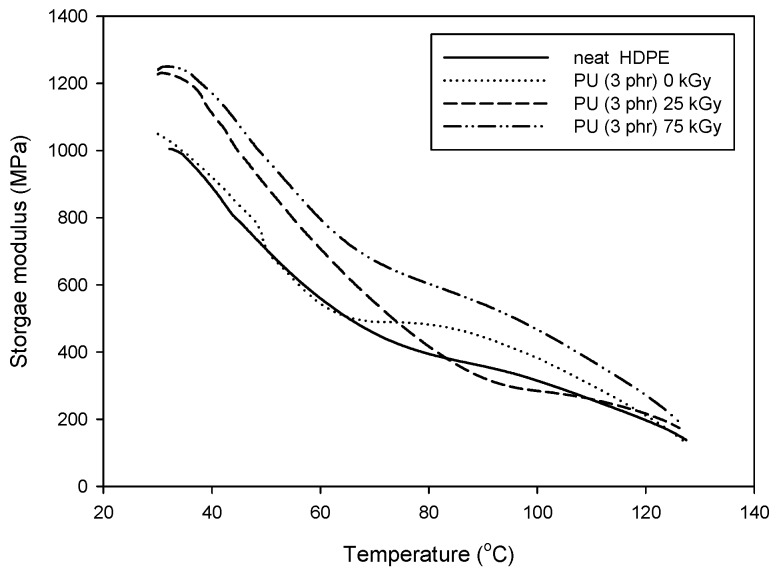
Storage modulus of PU (3 phr)/HDPE composite at various absorption doses.

The thermal-mechanical properties of the samples were measured using a DMA, which was varied from room temperature to 130 °C at a heating rate of 5 °C/min. The temperature dependencies of the storage modulus of the PU/HDPE composites at various absorption doses are shown in Figure 5. The storage modulus increased when the absorbed dose increased. As a results of the radiation-induced grafting, the irradiated PU/HDPE composite likely developed a stronger interfacial bond between the PU and the HDPE compared with non-irradiated PU/HDPE composites.

**Figure 6:**
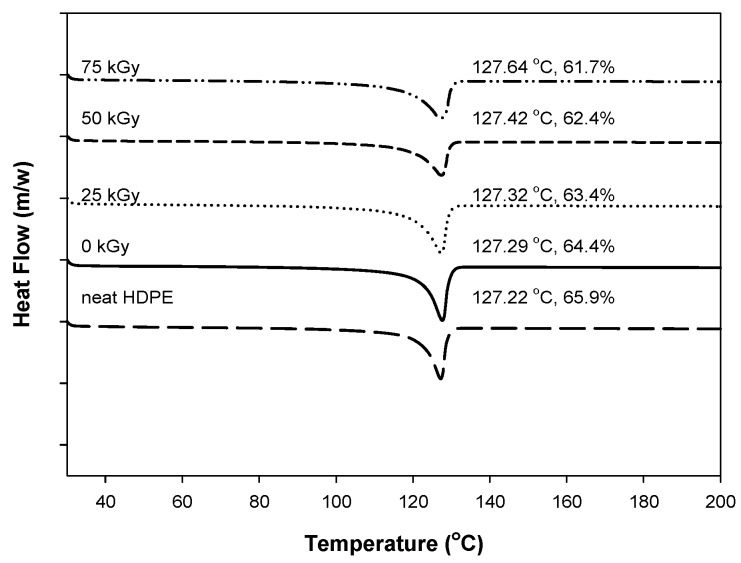
Differential scanning calorimetry (DSC) micrographs of PU (3 phr)/HDPE composites at various absorption doses.

Figure 6 shows the DSC heating thermograms of the PU/HDPE composites at various absorption doses under a heating rate of 10 °C/min. The melting peak at approximately 127°–128° corresponds to the melting temperature of the HDPE. The melting temperature slightly increased with increasing the radiation doses. The melting temperature increased after radiation-induced grafting reaction. However, the crystallinity decreased with increasing radiation dose. As shown in Figure 5, the crystallinity at 0 kGy was 64.4%, whereas at 75 kGy, it decreased to 61.7%. It was probably due to the grafted branches, which disrupted the regularity of the chain structure. Thus, the irregular structures formed through the grafting process reduce the crystallinity of HDPE molecular chains [13,17,18].

## 4. Conclusions

To reduce the environmental impact of industrial waste PU, the alternative recycling and reuse of PU is very important. In this study, we explored the utilization of waste PU as polymer filler via radiation-induced grafting. The grafting of waste PU onto HDPE matrices was carried out with one process using radiation with MAH. The radiation-induced grafting promoted the interfacial adhesion of PU onto HDPE matrices. When the absorbed dose was 75 kGy, the surface morphologies of the irradiated PU/HDPE composites presented as a smooth and single phase, and the elongation at break increased by approximately three-fold compared with that of non-irradiated PU/HDPE composites. Furthermore, the ductility of the HDPE composite was improved due to the successful radiation-induced grafting of waste PU onto the HDPE matrix. The improved properties suggest that HDPE/PU composite can be a good candidate for applications in high performance engineering, membranes for lithium ion batteries, and packaging.
